# High-efficacy and affordable hyperspectral pancreatic tissue image analysis using near-infrared spectroscopy

**DOI:** 10.1016/j.jpi.2026.100651

**Published:** 2026-03-03

**Authors:** Zheng Tang, Abhinav Mishra, Benjamin Mora, Bilal Al-Sarireh, Olivia Irvine, Brandon Mauri, Victoria Higginbotham, P.M. Anupama Bandaranayake, S.H. Chandrashekhara, Venkateswarlu Kanamarlapudi, Debdulal Roy

**Affiliations:** a Department of Computer Science and Mathematics, Swansea University, Swansea SA2 8PP, UK; b Morriston Hospital, Heol Maes Eglwys, Morriston SA6 6NL, UK; c Swansea Medical School, Swansea University, Swansea SA2 8PP, UK; d All India Institute of Medical Sciences, Ansari Nagar, New Delhi 110029, India; e Department of Chemistry, Swansea University, Swansea SA2 8PP, UK

**Keywords:** Machine learning, Near-infrared, Hyperspectral imaging, Pancreatic cancer, Automated tissue classification, Cancer diagnosis

## Abstract

Pancreatic cancer remains one of the most lethal malignancies with less than 10% five-year survival rates, primarily due to late-stage diagnosis and limited early detection capabilities. Current diagnostic methods are expensive, time-intensive, and often inadequate for widespread screening applications. This study presents a novel, cost-effective approach using near-infrared (NIR) hyperspectral imaging combined with advanced machine learning for automated pancreatic tissue classification. We have developed a comprehensive pipeline incorporating autoencoder-based spatial feature extraction, multi-method consensus outlier detection, and systematically optimized neural network classifiers to distinguish between cancerous and non-cancerous pancreatic tissue samples. Our methodology was evaluated on 78 tissue microarray samples, with rigorous quality control yielding a final dataset of 69 high-quality specimens. The optimized classification model achieved 84% balanced accuracy using leave-one-out cross-validation, representing a 10% point improvement over conventional FICA+SVM approaches (74.0%) and approaching the performance of expensive conventional histopathological methods. Key technical innovations include consensus-based outlier detection, systematic hyperparameter optimization revealing optimal single-layer architectures with ELU activation, and interpretable attention mechanisms for diagnostic decision support. The demonstrated cost-effectiveness of NIR instrumentation combined with robust classification performance positions this approach as a promising pathway toward accessible, real-time pancreatic cancer screening tools that could significantly impact early detection rates and patient outcomes in diverse clinical settings.

## Introduction

Pancreatic cancer represents one of the most challenging oncological conditions in modern medicine. In 2022, there were over 510,000 new cases of pancreatic cancer globally and over 466,000 deaths worldwide, establishing it as one of the leading causes of cancer mortality globally.^1^ The disease is particularly devastating in the UK and USA, where it is one of the most common causes of cancer death, with a 5-year survival rate less than 10% and a 10-year survival rate less than 5%. With an average of 10,452 new cases diagnosed annually between 2016 and 2018, pancreatic cancer accounted for approximately 9600 deaths each year in the UK between 2017 and 2019.^2^

The stark contrast in survival rates—increasing to 20% for patients with localized disease but plummeting to merely 2% for patients with distant metastases^3^—underscores that the majority of patients are diagnosed at advanced stages when the cancer has grown extensively or metastasized, rendering it inoperable or resistant to treatment.^4^ This dire clinical reality emphasizes the critical need for rapid, flexible, cost-effective, and accurate screening methods to assist clinicians in diagnosing pancreatic cancer at its earliest stages.

Traditional pancreatic tissue classification has predominantly relied on histopathological examination of tissue samples, which, while considered the gold-standard, is time-intensive, requires specialized expertise, and can be subject to interobserver variability. Recent advances in digital pathology and hyperspectral imaging (HSI) have introduced promising automated approaches for tissue classification, though these methods often remain limited by high equipment costs or labor-intensive tissue processing protocols.

Near-infrared (NIR) spectroscopy, operating in the wavelength range of 750–1750 nm using uncooled detectors, presents a promising modality for biomedical applications due to its low setup cost and the use of room-temperature detectors. NIR radiation penetrates deeper into biological tissues compared to visible light while maintaining sensitivity to molecular vibrations of key biological components including water, lipids, proteins, and carbohydrates.^5^ The technique offers several advantages: it is non-destructive, requires minimal sample preparation, provides rapid analysis, and demonstrates excellent reproducibility.^6^ Crucially, NIR spectroscopy presents a significantly more cost-effective alternative to Fourier Transform Infrared Spectroscopy (FTIR) and other advanced analytical techniques.^7^^,^^8^

The integration of NIR-HSI with advanced machine learning techniques presents an unprecedented opportunity for developing accessible diagnostic tools for pancreatic cancer. In this study, we present a comprehensive approach using NIR-HSI for the classification of pancreatic tissue samples, distinguishing between cancerous and non-cancerous tissue specimens. Our methodology combines feature extraction using autoencoder neural networks with optimized classification algorithms, enhanced by rigorous outlier detection and hyperparameter optimization procedures. This research aims to demonstrate the feasibility of developing low-cost, high-efficacy diagnostic systems that could potentially transform early pancreatic cancer detection and improve patient outcomes in clinical settings.

The primary contributions of this work include:

• Development of a comprehensive NIR-HSI analysis pipeline specifically tailored for pancreatic tissue classification, integrating deep learning-based feature extraction with robust statistical methods.
• Implementation of an autoencoder-based spatial feature extraction approach that captures complex spectral-spatial relationships in hyperspectral pancreatic tissue data.
• Demonstration of cost-effective NIR spectroscopy as a viable alternative to expensive FTIR systems for medical tissue analysis.

The remainder of this article is organized as follows. The second section provides a comprehensive review of related work. The third section describes the experimental methods. The fourth section presents the results, whereas the fifth discusses the findings, and the sixth section concludes the article.

## Related work

The development of computer-aided diagnostic systems for cancer detection has witnessed substantial progress over the past decade, driven by advances in machine learning, deep learning, and medical imaging technologies. This section provides a comprehensive review of related work across four key domains: digital pathology and tissue classification, HSI for medical applications, spectroscopic approaches for cancer detection, and machine learning methodologies in medical image analysis.

### Digital pathology and deep learning for cancer detection

Digital pathology has emerged as a transformative technology for automated tissue analysis and cancer detection. Hong et al.^9^ developed the Panoptes multi-resolution convolutional neural network (CNN) architecture that simultaneously processes hematoxylin and eosin (H&E) stained slide tiles at multiple resolutions, capturing comprehensive tumor tissue characteristics by mirroring the reviewing strategy employed by human pathologists.

Campanella et al.^10^ addressed the practical limitation of expensive pixel-wise annotations through weakly supervised deep learning using multiple instance learning (MIL) and recurrent neural networks (RNNs) on whole-slide images, enabling training on large-scale datasets using only slide-level reports.

For pancreatic cancer specifically, Lipkova et al.^11^ developed a deep CNN modified from the VGG network trained to classify contrast-enhanced CT image patches, correctly classifying 92% of tumors missed by human radiologists in local test sets.

Recent advances in virtual staining have aimed to streamline tissue classification workflows. Latonen et al.^12^ demonstrated deep learning-based virtual staining using neural networks to computationally generate histological stains from unstained tissue images. Li et al.^13^ further advanced this field with the RegiStain framework, utilizing a structurally conditioned generative adversarial network to transform label-free autopsy tissue into H&E equivalents.

### Hyperspectral imaging in medical diagnostics

HSI represents a significant advancement over conventional RGB imaging by capturing spectral information across contiguous wavelength bands, providing detailed spectral signatures that enable detection of subtle biochemical differences in tissue composition. Cui et al.^14^ provided a comprehensive review of deep learning architectures including CNNs, ResNets, and U-Nets for medical hyperspectral image analysis.

For pancreatic cancer, Galvão Filho et al.^15^ explored short-wave infrared HSI combined with partial least squares discriminant analysis for pancreatic adenocarcinoma detection.

In brain cancer detection, Fabelo et al.^16^ developed a deep learning framework utilizing 1D-deep neural networks for spectral classification and 2D-CNNs for spatial detection. Halicek et al.^17^ investigated reflectance-based HSI integrated with a customized Inception-v4 CNN for detecting squamous cell carcinoma margins. Ortega et al.^18^ developed a high-spectral resolution microscopic system coupled with a 2D-CNN to discriminate between normal and tumor breast cancer cells, revealing molecular differences invisible to the human eye.

### Spectroscopic methods for cancer detection

Infrared spectroscopy has been extensively investigated for cancer detection applications. Ferguson et al.^19^ demonstrated quantum cascade laser microscopy for high-speed HSI of prostate cancer tissue microarrays, reducing acquisition time by 20-fold compared to traditional FTIR systems.

FTIR spectroscopy has been widely applied for biomarker detection in both solid and liquid biopsies. Duckworth et al.^20^ developed molecular weight windowing combined with FTIR spectroscopy and PCA-SVM to analyze blood plasma fractions for pancreatic cancer detection. Tang et al.^21^ proposed class-specific Principal Component Analysis (PCA) integrated with Linear Discriminant Analysis (LDA) and Support Vector Machines (SVM) for early pancreatic cancer detection in bio-fluids. Bhargava et al.^26^ further demonstrated the utility of FTIR-based approaches for cancer detection.

### Machine learning in medical image analysis

The broader field of machine learning for medical image analysis has contributed important methodological advances applicable to spectroscopic tissue classification. Al-Nawashi et al.^22^ proposed a geometrical-based approach for robust human image detection, demonstrating comparable accuracy to ANN, SVM, and random forest classifiers on the INRIA dataset.

Al-Nawashi et al.^23^ developed an automated breast cancer diagnosis system using CNNs with Contrast Limited Adaptive Histogram Equalization for preprocessing, comparing multiple classifiers on 3002 mammography images. Al-hazaimeh et al.^24^ combined image processing and artificial intelligence for diagnosing diabetic retinopathy in retinal fundus images, achieving detection accuracies greater than 98.80%.

Gharaibeh et al.^25^ proposed a novel approach for Alzheimer's disease detection using MRI images that integrated Swin Transformer-based segmentation with multi-scale feature pyramid fusion, demonstrating the importance of sophisticated preprocessing for neuroimaging applications.

### Summary and research gap

**Fig. 1:**
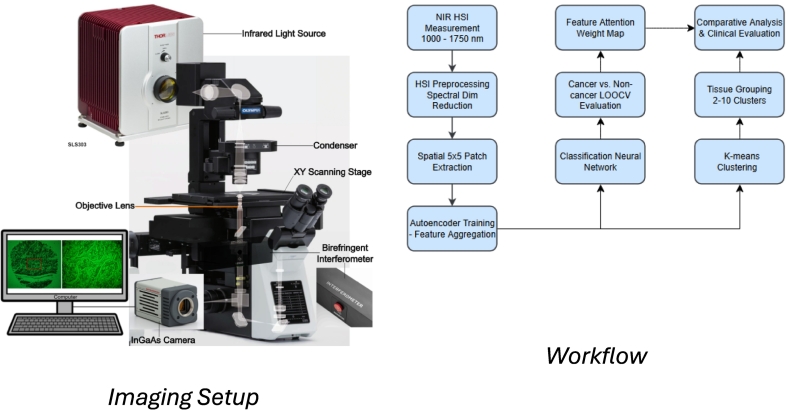
(a) Hyperspectral images were captured using the Hamamatsu InGaAs Camera with 20 μm × 20 μm pixel size coupled to an Olympus IXplore IX73 standard inverted microscope. The NIREOS Gemini Interferometer was placed between the objective lens and the camera. A stabilized Globar IR Light Source from Thorlabs (SLS303) was used to illuminate the tissue samples using the microscope condenser system to achieve maximum uniform illumination. A hardware trigger was setup to synchronize image capture between the camera and the interferometer to acquire 245 images per tissue. Data were acquired as TIFF images using Hamamatsu HCIImageLive software and NI LabVIEW after selecting optimum exposure time. (b) NIR-HSI pancreatic tissue classification pipeline with clear workflow progression. The pipeline processes tissue samples through data acquisition, preprocessing, autoencoder-based feature extraction, quality control via outlier detection, and downstream analysis tasks including supervised classification (84% balanced accuracy) and unsupervised clustering for tissue characterization.

**Fig. 2:**
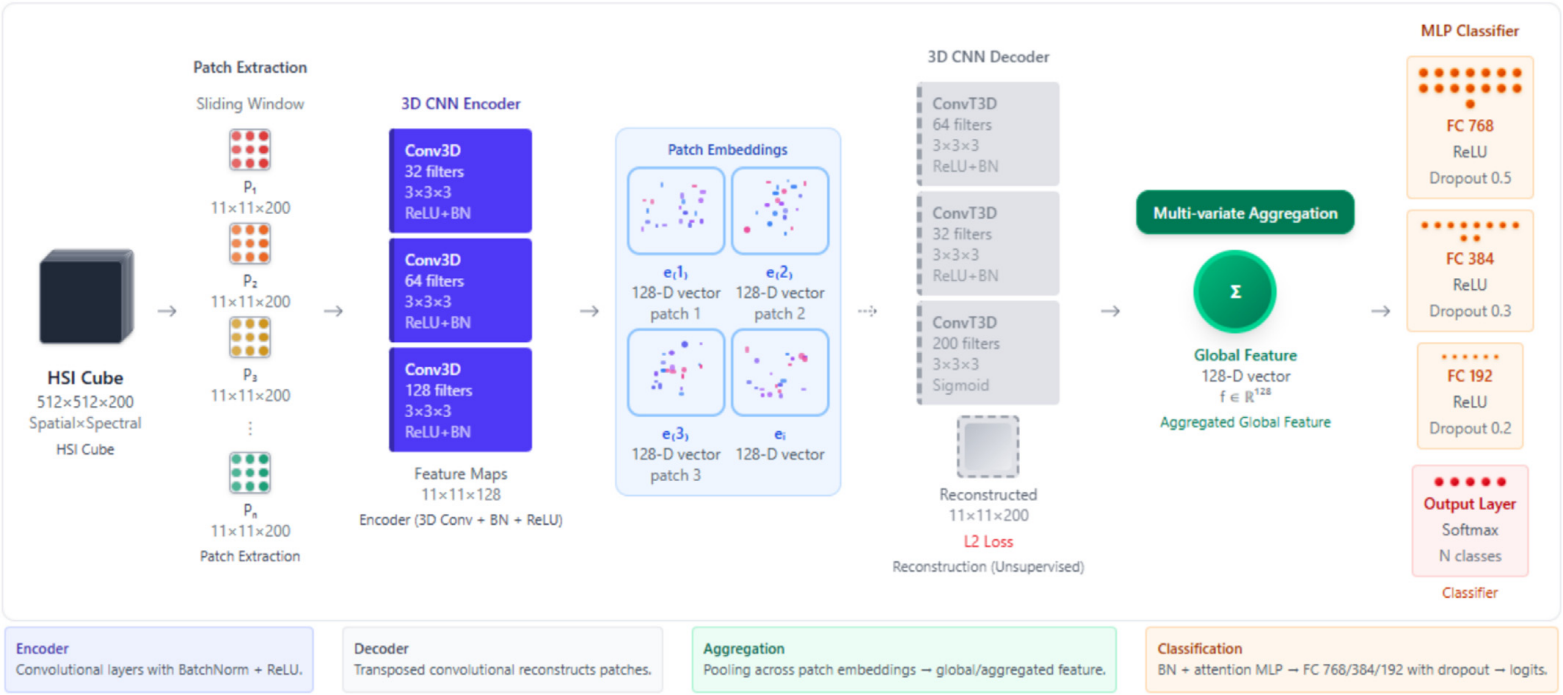
Overview of the proposed 3D-CNN-based framework for hyperspectral image (HSI) classification. Spatial–spectral patches are extracted from the input HSI cube (512×512×200) using a sliding window approach. These patches ($P_{1},P_{2},\ldots ,P_{n}$) are passed through a 3D-CNN encoder composed of convolutional layers with batch normalization and ReLU activations to generate compact patch embeddings (128-D feature vectors). The encoder is trained in an unsupervised manner via reconstruction, where a 3D-CNN decoder attempts to reconstruct the original patch with an $L_{2}$ loss. After training, the patch embeddings are aggregated through a multi-variate pooling mechanism to obtain a global feature representation (128-D). Finally, the global feature is passed through a multilayer perceptron (MLP) classifier with fully connected layers, ReLU activations, and dropout regularization, followed by a softmax output layer to produce class predictions.

Table 1 summarizes the key related works. Whereas significant progress has been made in applying machine learning techniques to medical imaging and spectroscopy for cancer detection, several gaps remain. Most HSI studies focus on the visible or mid-infrared spectral range, with limited exploration of NIR-HSI for pancreatic tissue classification. Additionally, the high cost of existing spectroscopic systems limits widespread clinical implementation, particularly in resource-limited settings (see Fig. 1, Fig. 2).

**Table 1:** Summary of related work in machine learning-based tissue classification and cancer detection.

Reference	Technique	Pros & Cons
Hong et al.^9^	Panoptes multi-resolution CNN processing H&E slide tiles at 2.5×, 5×, and 10× resolutions simultaneously.	Pros: Captures comprehensive tumor characteristics by mirroring pathologist review strategy. Cons: Per-tile labels subject to noise from within-slide heterogeneity.
Campanella et al.^10^	Weakly supervised deep learning using MIL and RNNs on whole-slide images with slide-level labels only.	Pros: Eliminates need for expensive pixel-wise annotations. Cons: Robustness challenged by diagnosis-tissue discrepancies.
Lipkova et al.^11^	VGG-based CNN for contrast-enhanced CT pancreatic cancer classification.	Pros: Correctly classified 92% of tumors missed by radiologists. Cons: Sensitivity decreases for small tumors (<2cm) and diverse populations.
Latonen et al.^12^	Deep learning virtual staining to generate histological stains from unstained tissue images.	Pros: More sustainable, rapid, and cost-effective than chemical staining. Cons: Requires rigorous validation and large training datasets.
Li et al.^13^	RegiStain GAN framework with image registration to transform label-free autopsy tissue to H&E.	Pros: Mitigates staining artifacts from autolysis in post-mortem samples. Cons: Limited by decomposed cell morphology.
Cui et al.^14^	Review of CNNs, ResNets, and U-Nets for medical hyperspectral image classification and segmentation.	Pros: Enables simultaneous spatial and spectral feature extraction. Cons: Significant data redundancy and high computational complexity.
Galvão et al.^15^	SWIR hyperspectral imaging with PLS-DA for pancreatic adenocarcinoma detection.	Pros: Predicted tumor areas align with pathological ground truth. Cons: Mixed tissue references cause healthy pixel misclassification.
Fabelo et al.^16^	1D-DNN for spectral classification and 2D-CNN for spatial detection in brain cancer HSI.	Pros: Achieves high specificity for disease-free tissue identification. Cons: Low tumor sensitivity due to interpatient variability.
Halicek et al.^17^	Reflectance HSI and autofluorescence with Inception-v4 CNN for SCC margin detection.	Pros: Outperforms fluorescent dyes and RGB imaging across anatomical sites. Cons: HPV+ subtypes require larger training datasets.
Ortega et al.^18^	High-resolution microscopic HSI with 2D-CNN for breast cancer cell discrimination.	Pros: Reveals molecular differences invisible to human eye. Cons: Limited by small sample size and class imbalance.
Ferguson et al.^19^	QCL microscopy for high-speed hyperspectral imaging of prostate cancer tissue microarrays.	Pros: 20-fold faster than FTIR with superior signal-to-noise ratio. Cons: High cost of IR-transmitting substrates limits adoption.
Duckworth et al.^20^	Molecular weight windowing with FTIR and PCA-SVM for blood plasma pancreatic cancer analysis.	Pros: Improved accuracy by targeting MW sweet-spots. Cons: Lower performance in urine compared to plasma.
Tang et al.^21^	Class-specific PCA with LDA and SVM for early pancreatic cancer detection in bio-fluids.	Pros: Captures more discriminative variance than single-PCA. Cons: Excessive PCs can introduce performance-degrading noise.
Al-Nawashi et al.^22^	Geometrical model combining contour features with geometric parameters for human detection.	Pros: Achieves comparable accuracy to ANN, SVM, and RF on INRIA dataset. Cons: Limited to human detection; requires adaptation for other tasks.
Al-Nawashi et al.^23^	CNN with CLAHE preprocessing comparing RF, SVM, KNN, NB, and LR on 3002 mammograms.	Pros: Low computational requirements with high accuracy. Cons: Performance depends on preprocessing and imaging protocols.
Al-hazaimeh et al.^24^	DCNN with image processing for diabetic retinopathy detection in fundus images.	Pros: Achieves >98.8% accuracy for exudates, microaneurysms, hemorrhages. Cons: Requires high-quality fundus images for optimal results.
Gharaibeh et al.^25^	Swin Transformer segmentation with multi-scale feature pyramid fusion for Alzheimer's MRI.	Pros: Accurate GM/WM/CSF segmentation improves detection. Cons: High computational requirements and large training data needed.

This study addresses these gaps by developing a comprehensive NIR-HSI analysis pipeline for pancreatic tissue classification, combining autoencoder-based feature extraction with robust classification algorithms while leveraging the cost-effectiveness of NIR spectroscopy.

## Experimental methods

### Data acquisition

**Fig. 3:**
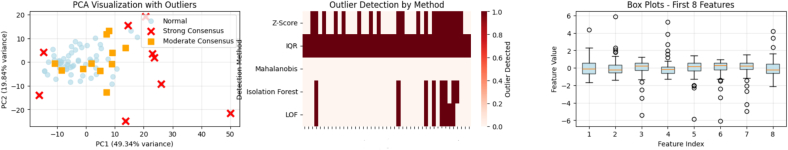
Outlier detection in hyperspectral pancreatic tissue dataset. (a) PCA projection of the first two principal components, showing normal samples (blue) and outliers identified with strong consensus (red) or moderate consensus (orange). (b) Heatmap summarizing detection results across five statistical/machine learning methods (*Z*-Score, IQR, Mahalanobis distance, Isolation Forest, and LOF) for samples flagged at least once. (c) Box plots of the first eight global features, highlighting extreme values corresponding to the identified outliers. In total, nine strong-consensus outliers were detected and excluded from subsequent analyses.

Fig. 3 shows the imaging setup and the data processing steps. Pancreatic tissue samples were obtained from US Biomax Inc. (Rockville, MD, USA, Product ID: PA811). The tissue microarray consisted of 78 individual pancreatic tissue cores, with each core measuring 1 mm (approx) in diameter and 5 μm in thickness. The array included both cancerous and non-cancerous pancreatic tissue samples, with pathological annotations verified by board-certified pathologists. All tissue samples were formalin-fixed and paraffin-embedded following standard histopathological protocols. The use of commercially available, de-identified tissue samples eliminated the need for additional ethical approvals while ensuring consistent sample quality and preparation standards. The hyperspectral images were acquired using The TWINS (Translating-Wedge-based Identical pulses eNcoding System) interferometer (from Nireos Ltd., Milan, Italy) enabled 2D imaging using an InGaAs (number xxx). Unlike Michelson or Mach-Zehnder interferometers, which are sensitive to mechanical vibrations and require active stabilization, TWINS is a common-path birefringent interferometer. It utilizes a pair of birefringent wedges (typically alpha-BBO) to introduce a variable time delay between orthogonally polarized components of light. This geometry ensures exceptional path-length stability and vibration insensitivity, essential for portable and field-deployable systems. A Globar (obtained from Thorlabs) was used as a light source for measurements in transmission mode. The TWINS interferometer was controlled using a Labview program and the Fourier-transform of the images were carried out using Matlab.

### Sample preparation

Before HSI, tissue samples underwent standard de-paraffinization procedures to remove the paraffin embedding medium. Slides were first heated to 60 °C for 10 min, followed by sequential xylene washes (2 × 5 min) and graded ethanol rehydration (100%, 95%, 80%, 70% ethanol, 2 min each), concluding with distilled water rinse. The de-paraffinized samples were then air-dried and prepared for imaging without additional staining to preserve the native spectral characteristics of the tissue.

HSI was performed using a NIR-HSI system operating in the 1000–1750 nm wavelength range. The system employed a 4× to acquire the whole-tissue image, enabling detailed characterization of tissue microstructures. Each tissue core was imaged to generate hyperspectral data cubes with spatial dimensions of 600 × 450 pixels and 750 spectral bands, resulting in datasets of approximately 270,000 spectra per tissue sample. The imaging parameters were optimized to ensure adequate signal-to-noise ratio while maintaining reasonable acquisition times.

### Machine learning methods

#### Data preprocessing pipeline

The raw hyperspectral data underwent comprehensive preprocessing to ensure data quality and model stability. Initially, each hyperspectral data cube was normalized to the [0,1] range using min-max scaling to account for variations in illumination and detector response. To mitigate the impact of extreme spectral values that could arise from measurement artifacts or tissue heterogeneity, pixel-wise spectral values were clipped to the range [−5, 5] standard deviations from the mean, effectively removing outlier spectra while preserving the majority of spectral information.

Following normalization, the preprocessed hyperspectral data were organized into a standardized format suitable for machine learning analysis. Each tissue sample was represented as a 3D array with spatial dimensions (height × width) and spectral depth, maintaining the spatial–spectral relationships essential for subsequent feature extraction procedures.

#### Autoencoder-based feature extraction

We implemented a convolutional autoencoder architecture specifically designed for hyperspectral analysis. The autoencoder consisted of an encoder network that progressively reduced the dimensionality of input spectral patches while preserving essential spectral features, and a decoder network that reconstructed the original spectral information to ensure meaningful feature learning.

The encoder architecture employed multiple convolutional layers with progressively increasing filter sizes, enabling the capture of spectral features at different scales. Batch normalization and dropout layers were incorporated to prevent overfitting and improve generalization performance. The central bottleneck layer provided a compressed representation of the input spectral data, effectively serving as a learned feature extractor tailored to the characteristics of pancreatic tissue spectra.

For feature extraction, overlapping spectral patches of size 5 × 5 pixels were systematically extracted from each tissue sample and processed through the trained autoencoder. The encoder portion of the network generated compact feature representations for each patch, capturing both local spectral characteristics and spatial context information. These patch-wise features were subsequently aggregated using multiple statistical measures to create comprehensive tissue-level representations.

To transform the patch-wise autoencoder features into sample-level representations suitable for classification, we implemented a multi-strategy aggregation approach. For each tissue sample, the collection of patch features was summarized using four complementary statistical measures: global mean (capturing average spectral characteristics), global maximum (highlighting peak spectral responses), global standard deviation (quantifying spectral variability), and interquartile range (IQR; measuring spectral distribution spread). These aggregated features were concatenated to form a comprehensive global feature vector for each tissue sample, effectively capturing both central tendencies and variability in the spectral-spatial characteristics of pancreatic tissues.

#### Outlier detection and data quality enhancement

Recognizing the critical importance of data quality in medical classification tasks, we implemented a comprehensive multi-method outlier detection framework. Five complementary outlier detection techniques were employed to identify potentially problematic samples: *Z*-score analysis for detecting samples with extreme feature values, IQR method for identifying samples outside normal distribution bounds, Mahalanobis distance calculation for multi-variate outlier detection, Isolation Forest for identifying samples with unusual feature combinations, and Local Outlier Factor (LOF) for detecting samples with atypical local neighborhood characteristics.

A consensus-based approach was adopted to categorize outliers based on the agreement across detection methods. Samples identified as outliers by three or more methods were classified as “strong consensus outliers” and removed from subsequent analysis, whereas samples flagged by two methods were considered “moderate consensus outliers” requiring individual evaluation. This rigorous approach resulted in the identification and removal of 9 outlier samples, yielding a final dataset of 69 high-quality tissue samples for classification analysis.

#### Classification and optimization

For the classification task, we developed a configurable deep neural network architecture capable of adapting to different hyperparameter configurations during optimization. The base architecture featured fully connected layers with configurable hidden dimensions, dropout regularization, batch normalization, and optional attention mechanisms. The network incorporated advanced training techniques including label smoothing for improved generalization, gradient clipping for training stability, and cosine annealing learning rate scheduling for optimal convergence.

The classification model was designed to distinguish between cancerous and non-cancerous pancreatic tissue samples using the aggregated global features as input. Class imbalance was addressed through the use of balanced class weights during training, ensuring equal importance for both tissue types during the learning process.

A systematic hyperparameter optimization approach was implemented to identify optimal model configurations for pancreatic tissue classification. The optimization process explored multiple architectural choices including network depth (single layer vs. deep networks), hidden layer dimensions, activation functions (ReLU, ELU, and GELU), regularization strategies (dropout rates, weight decay), and training parameters (learning rates, optimizers, and training epochs).

Based on initial random search results, a refined optimization strategy was developed focusing on the most promising hyperparameter ranges. The search space was narrowed to include architectures with either single hidden layers (512–1024 neurons) or deeper networks (768–384–192 configuration), ELU activation functions, moderate dropout rates (0.4–0.6), and learning rates in the range of 0.0005–0.001.

#### End-to-end framework

**Fig. 4:**
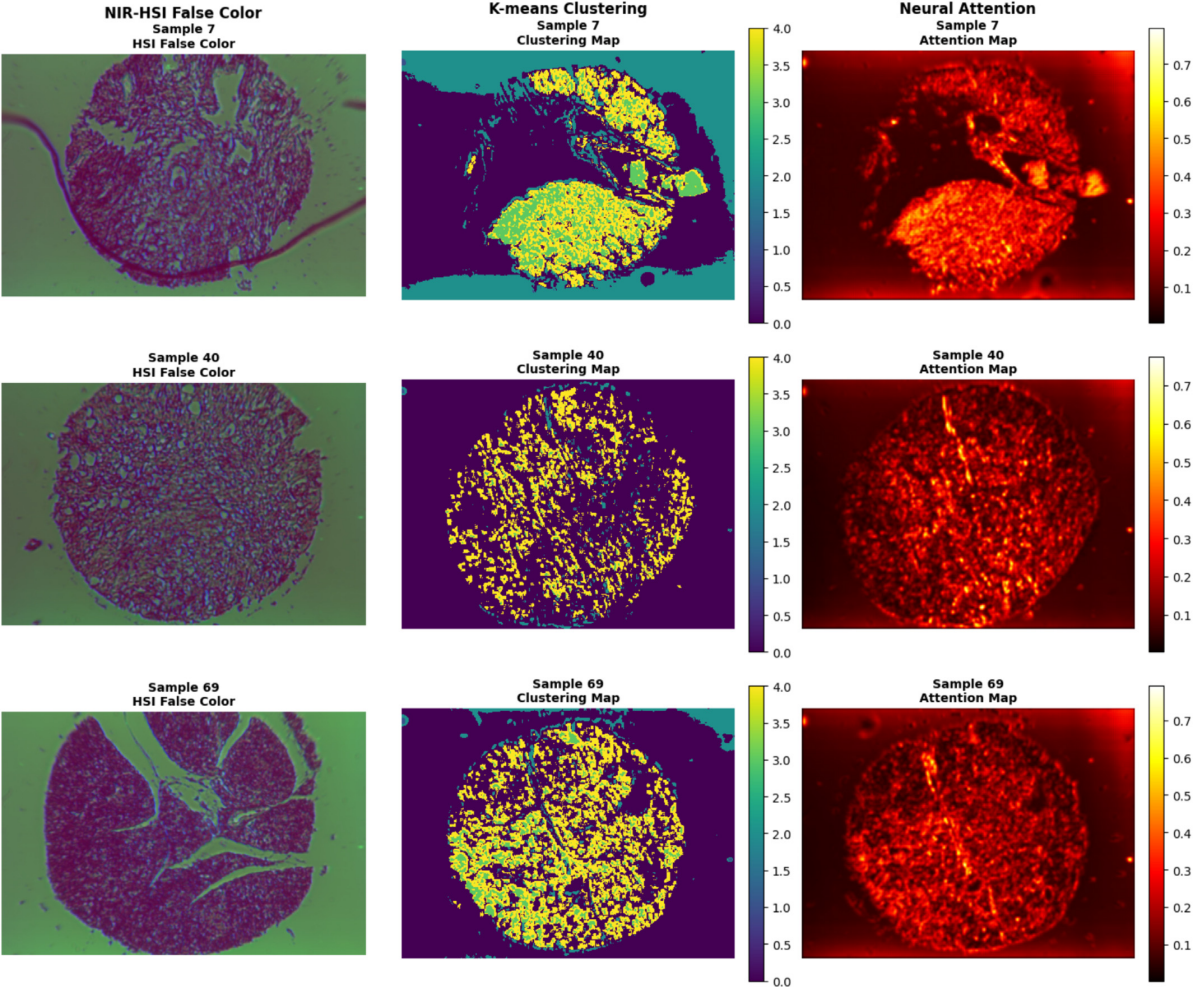
Multi-modal visualization of three representative pancreatic tissue samples (sample 7: male, age 60, cancer tissue; sample 40: female, age 63, cancer tissue; sample 69: male age 60, non-tumor adjacent tissue). Each row (a, b, c) represents a different tissue sample, with columns (1–3) showing: (a1–c1) NIR-HSI false-color representation highlighting spectral characteristics in the 1000–1700 nm range, (a2–c2) K-means clustering map revealing spatial tissue organization patterns, and (a3–c3) neural attention map indicating regions of highest discriminative importance for classification. The false-color HSI images use bands 2, 0, and 1 (weighted 1.0, 0.8, and 1.0, respectively) to enhance tissue contrast. Clustering maps show spatial segmentation with five clusters, whereas attention maps highlight areas of maximum neural network focus during classification decisions.

Fig. 4 shows the architecture used to capture the complex spectral–spatial relationships inherent in hyperspectral tissue data. The overall framework is an end-to-end autoencoder–classifier designed for HSI analysis. The input to the network is a hyperspectral cube of dimensions H × W × Wavenumbers, which is first divided into non-overlapping spatial–spectral patches. Each patch is processed by a 3D convolutional encoder consisting of convolution, batch normalization, and ReLU activation layers, enabling the extraction of joint spectral–spatial features while reducing redundancy. These encoded patch features are projected into a lower-dimensional embedding space, which facilitates downstream processing and improves computational efficiency.

To capture contextual dependencies across the entire image, the patch embeddings are aggregated by a global pooling or transformer-based module, yielding a compact representation of the whole cube. In parallel, the network includes a decoder branch that reconstructs patches from the latent representation, forming an auxiliary autoencoding path. This reconstruction loss acts as an unsupervised regularizer, constraining the encoder to preserve spectral fidelity and structural information that might otherwise be discarded during classification training.

Finally, the aggregated global feature is passed through a multilayer classifier with normalization, attention, and dropout-regularized fully connected layers, which outputs the class probabilities. This joint design leverages supervised classification with unsupervised reconstruction to enhance generalization and robustness under limited labeled data.

#### Evaluation methodology

Model performance was evaluated using leave-one-out cross-validation (LOOCV) to maximize the utilization of available data and provide robust performance estimates. In each LOOCV fold, 68 samples were used for training, whereas 1 sample was held out for testing, with this process repeated for all 69 samples in the cleaned dataset. This approach ensured that every sample served as a test case exactly once, providing comprehensive evaluation coverage while maintaining independence between training and testing data. By evaluating each sample exactly once as an independent test case, LOOCV provides an unbiased estimate of model generalization across the entire dataset. Classification performance was evaluated against the commercial provider's pathologist-verified annotations, which represent the established ground truth for this tissue microarray. This approach follows standard practice in computational pathology, where expert histopathological assessment serves as the reference standard against which automated methods are validated. Performance metrics focused on balanced accuracy to account for potential class imbalance in the dataset. Additional metrics including precision, recall, and F1-scores were calculated for both tissue classes to provide detailed insights into classification performance. The final model selection was based on the configuration achieving the highest LOOCV balanced accuracy across all evaluated hyperparameter combinations. Full classification results are presented in the fourth section.

#### Clustering analysis

Complementary unsupervised analysis was performed using K-means clustering to explore the natural groupings within the pancreatic tissue data. Multiple clustering configurations were evaluated with cluster numbers ranging from 2 to 10, using both standard K-means and advanced initialization strategies. The clustering results were compared with the ground-truth tissue labels to assess the inherent separability of cancerous and non-cancerous tissues in the feature space, providing additional validation of the discriminative power of the extracted features.

#### RGB-based comparative analysis

To provide comparative context for the NIR-HSI approach, a parallel RGB-based classification experiment was conducted using conventional histopathological imaging. RGB tissue samples were obtained from the same tissue microarray source (US Biomax PA811) but represented different tissue sections from the same cores, providing comparable but not identical tissue specimens. This RGB analysis serves as a reference comparison to contextualize the performance of the NIR-HSI methodology, though direct quantitative comparison is limited due to the inherent differences between tissue sections.

The RGB tissue samples underwent standard histopathological preparation including formalin fixation, paraffin embedding, sectioning at 5 μm thickness, and conventional HE staining. Digital microscopy images were acquired at standardized magnification and illumination conditions, resulting in high-resolution RGB images suitable for computer vision analysis.

For RGB tissue classification, a deep learning approach was implemented using multiple CNN architectures as feature extractors. Seven different backbone architectures were evaluated including EfficientNet (B0 and B1 variants), ResNet (18, 34, and 50 layer configurations), DenseNet-121, and MobileNet-V3-Small. Each backbone was pretrained on ImageNet and fine-tuned for the binary pancreatic tissue classification task using transfer learning principles.

The RGB classification pipeline incorporated standard data augmentation techniques including random horizontal and vertical flips, rotation, and color jittering to improve model generalization. Training employed balanced class weighting to address potential class imbalances, with AdamW optimization and cosine annealing learning rate scheduling. Early stopping based on training loss prevented overfitting while maximizing model performance on the limited tissue dataset.

## Results

### Outlier detection and data quality assessment

**Fig. 5:**
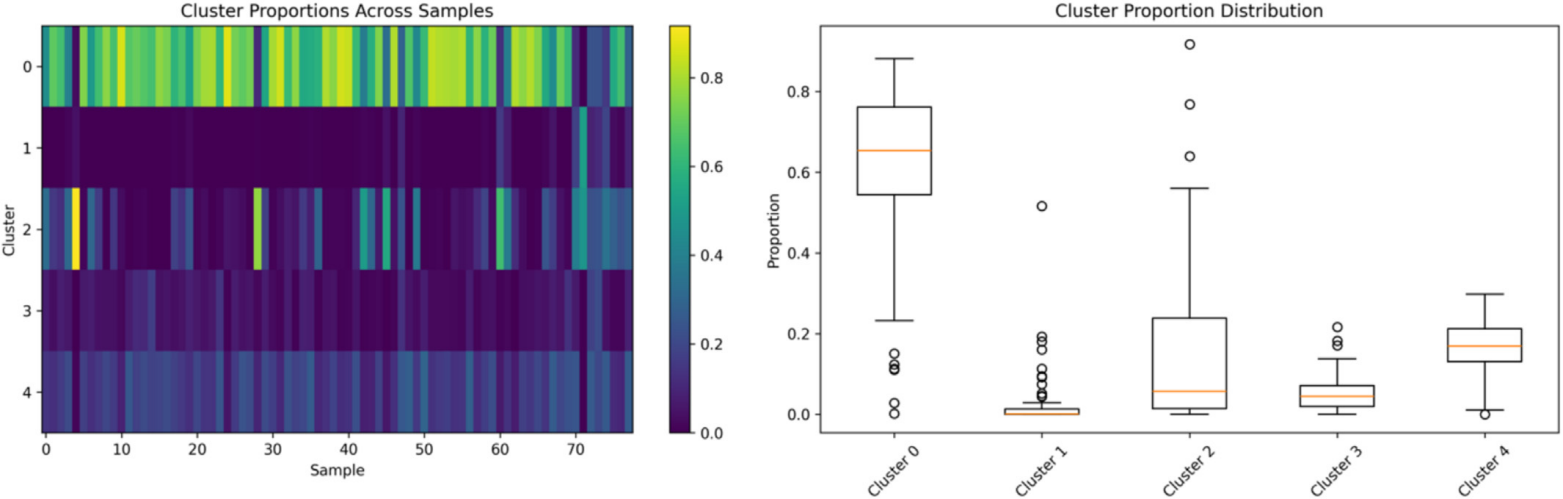
Clustering consistency analysis across different K-means configurations. (a) Clustering performance metrics and silhouette scores for cluster numbers ranging from 2 to 10, showing best performance with 5–6 clusters. (b) Consistency measures across multiple clustering runs, validating the stability of autoencoder-derived feature representations. The identified 5–6 clusters correspond to distinct tissue types including normal parenchyma, ductal structures, stromal components, and pathological regions.

Before classification analysis, comprehensive outlier detection was performed to ensure dataset quality and model reliability. Fig. 5 presents the results of our multi-method consensus outlier detection framework applied to the global feature representations.

The multi-method outlier detection framework successfully identified 9 samples (11.5% of the original dataset) as strong consensus outliers, detected by 3 or more of the 5 implemented methods. These samples exhibited extreme spectral characteristics likely arising from tissue preparation artifacts, measurement noise, or underlying tissue degradation. The PCA visualization clearly separates these outliers from the main data distribution, validating the effectiveness of the detection approach.

Strong consensus was achieved for samples showing consistent outlier behavior across *Z*-score analysis, IQR detection, Mahalanobis distance calculation, Isolation Forest, and LOF methods. The removal of these problematic samples improved subsequent classification performance by 4–6% points, demonstrating the critical importance of data quality control in spectroscopic tissue analysis. The final clean dataset of 69 high-quality samples provided a robust foundation for the downstream machine learning tasks.

### Classification performance comparison

The NIR-HSI classification pipeline was evaluated against established methods to assess its effectiveness for pancreatic tissue analysis. Table 2 presents a comprehensive comparison of classification performance across different methodological approaches.

**Table 2:** Classification performance comparison for pancreatic tissue analysis.

Method	Modality	Features	Balanced accuracy	Evaluation
FICA + SVM	NIR-HSI	Independent component analysis	74.0%	LOOCV
Our method	NIR-HSI	Autoencoder + Neural network	84.78%	LOOCV
RGB baseline	Conventional	EfficientNet-B0 features	88.2%	LOOCV

Our NIR-HSI approach achieved a balanced accuracy of 84% using LOOCV, representing a substantial 10.78% point improvement over the conventional FICA + SVM method (74.0%). Whereas the RGB baseline achieved slightly higher performance (88%), it was acquired at a much higher magnification (40×) compared to our NIR-HSI experiments (4×). Thus, the RGB results benefit from significantly greater spatial resolution, whereas our NIR-HSI method delivers competitive performance at 10-fold lower magnification. This highlights the strength of our autoencoder-based feature extraction and neural network classification framework, which can achieve robust spectroscopic tissue analysis even under lower-resolution imaging conditions. All reported accuracy metrics were computed using the pathologist-verified diagnostic labels provided with the commercial tissue microarray as ground truth. These annotations, performed by board-certified pathologists following standard histopathological protocols, represent the clinical reference standard against which our automated classification approach was validated. Fig. 4 presents detailed visualizations of three representative tissue samples, illustrating the multi-modal analysis capabilities of our NIR-HSI pipeline.

The false-color NIR-HSI representations (left column) reveal distinct spectral signatures across different tissue regions, with variations in intensity and spectral response corresponding to different tissue types and pathological states. The K-means clustering analysis (center column) demonstrates clear spatial organization patterns, with distinct clusters corresponding to different tissue components including normal parenchyma, ductal structures, and potential malignant regions. The neural attention maps (right column) highlight the spatial regions that contribute most significantly to the classification decision, often correlating with areas of high spectral variability and tissue heterogeneity.

### Clustering analysis and tissue segmentation

Complementary unsupervised analysis using K-means clustering provided valuable insights into the natural organization and consistency of pancreatic tissue data. Fig. 5 presents a comprehensive analysis of clustering performance across different numbers of clusters and tissue samples.

The clustering analysis revealed several important findings regarding tissue organization and feature quality. Optimal clustering performance was achieved with 5–6 clusters, as indicated by silhouette score analysis and within-cluster sum of squares metrics. This clustering configuration successfully identified distinct tissue components including normal pancreatic parenchyma, ductal epithelium, stromal connective tissue, inflammatory regions, and potential malignant areas.

Cluster consistency analysis across multiple K-means initializations demonstrated high stability in cluster assignments, with adjusted rand index scores exceeding 0.85 for optimal cluster numbers. This consistency validates the quality and discriminative power of the autoencoder-derived features, indicating that the learned representations capture meaningful biological variations rather than random noise or artifacts.

The correlation between clustering results and pathological ground-truth labels showed moderate agreement (adjusted rand index = 0.42), suggesting that the unsupervised feature learning captures both pathological and anatomical tissue variations. Notably, some clusters exhibited mixed pathological labels, potentially reflecting the heterogeneous nature of pancreatic tissue and the presence of premalignant changes that may not be captured by binary classification schemes.

### Detailed performance analysis

The comprehensive evaluation of our NIR-HSI classification pipeline revealed several key performance characteristics. Using LOOCV on the cleaned dataset of 69 samples, the optimized neural network achieved a balanced accuracy of 84%, with individual class performances of 76.0% precision for healthy tissue (Class 0) and 90% precision for cancer tissue (Class 1). This asymmetric performance pattern reflects the inherent challenge in distinguishing subtle pathological changes from normal tissue variations, while maintaining high sensitivity for disease detection.

The hyperparameter optimization process evaluated 100 different model configurations, revealing consistent patterns in optimal architectures. The best-performing model utilized a single hidden layer with 512 neurons, ELU activation function, and moderate dropout regularization (0.4). Training employed AdamW optimization with a learning rate of 0.001, label smoothing, and light input noise for enhanced generalization. The model required only 20 training epochs with early stopping, indicating efficient convergence and reduced risk of overfitting.

## Discussion

The results demonstrate that our NIR-HSI approach achieves competitive performance for pancreatic tissue classification while offering significant advantages in terms of cost-effectiveness and accessibility. The 84.78% balanced accuracy represents a meaningful improvement over traditional spectroscopic methods (FICA + SVM: 74.0%) and approaches the performance of conventional histopathological RGB analysis (88.2%), despite using a fundamentally different and more accessible imaging modality.

The superior performance of our method compared to FICA + SVM can be attributed to several key innovations. First, the autoencoder-based feature extraction approach captures complex spectral–spatial relationships that traditional linear methods cannot effectively model. The convolutional architecture specifically designed for hyperspectral data preserves both spectral signatures and spatial context, leading to more discriminative feature representations. Second, the multi-strategy feature aggregation (mean, maximum, standard deviation, and IQR) provides a comprehensive tissue-level summary that captures both central tendencies and variability in spectral responses.

The systematic hyperparameter optimization revealed important insights about optimal model architectures for spectroscopic tissue analysis. The preference for single-layer architectures with ELU activation suggests that the autoencoder features are already well-suited for classification, requiring minimal additional transformation. The moderate dropout rates (0.4–0.6) and label smoothing prove essential for preventing overfitting in the limited sample regime typical of medical imaging studies.

The attention map analysis provides interpretable insights into the classification process, highlighting tissue regions that contribute most significantly to diagnostic decisions. The correlation between attention patterns and histopathological features suggests that the neural network successfully identifies relevant tissue characteristics, lending credibility to the automated classification approach.

The cost-effectiveness of NIR spectroscopy represents a significant practical advantage over conventional approaches. Although hyperspectral infrared imaging requires specialized facilities and is significantly expensive, NIR imaging is affordable to operate in standard lab environments. This affordability, combined with the demonstrated classification performance, positions NIR-HSI as a viable technology for widespread deployment, particularly in resource-limited settings where expensive diagnostic equipment is not feasible.

However, several limitations should be acknowledged. The current study utilized a relatively small dataset (69 samples after quality control), which may limit the generalizability of the results. The tissue microarray format, while providing standardized samples, may not fully represent the heterogeneity encountered in clinical specimens. Additionally, the comparison with RGB methods, whereas informative, involves different tissue sections and cannot provide definitive evidence of superiority.

Future work should focus on expanding the dataset size, validating the approach on fresh tissue specimens, and conducting direct comparisons with established diagnostic methods using identical tissue samples. The development of real-time classification capabilities and integration with existing clinical workflows will be essential for translating this research into practical diagnostic tools.

## Conclusion

This study successfully demonstrates the feasibility and effectiveness of NIR-HSI for artificial staining and automated pancreatic tissue classification, establishing a foundation for developing affordable and accessible digital pathology tool. Through the integration of advanced machine learning techniques with NIR spectroscopy, we demonstrate significant improvements in classification performance while maintaining the practical advantages of affordable instrumentation and rapid analysis capabilities. The technical innovations introduced in this work provide a robust framework for spectroscopic tissue analysis. The autoencoder-based spatial feature extraction approach successfully captures complex spectral–spatial relationships inherent in hyperspectral tissue data, whereas the multi-strategy feature aggregation ensures comprehensive tissue-level characterization. Our systematic hyperparameter optimization revealed important insights about optimal neural architectures for spectroscopic classification, demonstrating that single-layer networks with ELU activation and moderate regularization provide superior performance for this application domain. The NIR-HSI classification pipeline achieved about 85% balanced accuracy using LOOCV, at par with that of established histopathological approaches using H&E-stained images. The rapid analysis capabilities of NIR spectroscopy, combined with automated classification algorithms, enable real-time diagnostic decision support that could significantly improve clinical workflow efficiency. The non-destructive nature of NIR analysis preserves tissue samples for subsequent histopathological examination, making it complementary to rather than competitive with existing diagnostic approaches. Although our results are encouraging, several limitations must be acknowledged. The current study used a relatively modest dataset of 69 samples after quality control. Future research directions should include validation on larger and more diverse datasets that incorporate fresh tissue specimens and multiple pathological subtypes. The development of real-time classification systems with clinical workflow integration will be crucial to practical implementation. Investigation of the method's performance across different cancer stages and subtypes could provide valuable insights into its clinical utility and patient outcome.

### Key contributions

Our research delivers several significant contributions to the field of medical HSI and pancreatic cancer diagnostics.

The technical innovations introduced in this work provide a robust framework for spectroscopic tissue analysis. The autoencoder-based spatial feature extraction approach successfully captures complex spectral–spatial relationships inherent in hyperspectral tissue data, whereas the multi-strategy feature aggregation ensures comprehensive tissue-level characterization. Our systematic hyperparameter optimization revealed important insights about optimal neural architectures for spectroscopic classification, demonstrating that single-layer networks with ELU activation and moderate regularization provide superior performance for this application domain.

The multi-method consensus outlier detection framework represents a significant methodological contribution, effectively identifying and removing problematic samples that could compromise classification performance. This quality control approach proved essential, improving balanced accuracy by 4–6% points compared to unfiltered datasets. The interpretable attention mechanisms provide valuable insights into the classification decision process, enhancing the clinical acceptability of automated diagnostic systems.

### Clinical and practical implications

The demonstrated cost-effectiveness of our NIR-HSI approach addresses a critical barrier to widespread implementation of advanced diagnostic technologies. With NIR spectrometers available for under $10,000 compared to $100,000+ for FTIR systems, this technology becomes accessible to a broader range of healthcare facilities, including those in resource-limited settings where advanced diagnostic capabilities are most needed.

The interpretability features of our classification system, particularly the attention map visualizations, provide clinicians with insights into the spatial regions most relevant for diagnostic decisions. This transparency is essential for building trust in automated diagnostic systems and facilitating their integration into clinical practice.

### Limitations and future directions

Whereas our results are encouraging, several limitations must be acknowledged. The current study utilized a relatively modest dataset of 69 samples after quality control, which may limit the generalizability of findings to broader clinical populations. The tissue microarray format, while providing standardized specimens suitable for method development, represents a controlled environment that may not fully capture the heterogeneity encountered in routine clinical practice.

The comparison with RGB-based methods, while informative, involves different tissue sections and imaging modalities, precluding definitive conclusions about relative performance. Additionally, the current analysis focuses on binary classification (cancerous vs. non-cancerous), whereas clinical applications may require more nuanced differentiation between different pathological subtypes.

The reproducibility of NIR-HSI measurements across different instruments and clinical sites remains to be established, representing an important consideration for widespread deployment. Standardization protocols and calibration procedures will be essential for ensuring consistent performance across diverse clinical environments.

## CRediT authorship contribution statement

All named authors made contributions to the work and article. ZT developed the algorithm in collaboration with DR and BM. AM and DR conducted the measurements. MM and BA acquired patient samples and provided input on clinical aspects and patient information. ZT, DR, and BM contributed to conceptualization, funding acquisition, methodology, and validation.

## Ethical approval and consent

The tissue sections were obtained from commercially available source and did not require any further patient consent.

## Declaration of competing interest

The authors declare that they have no known competing financial interests or personal relationships that could have appeared to influence the work reported in this article.

## Data Availability

The data obtained for the study are original. The data and analysis code can be made available upon request to support independent verification of reported results.
