# Supplementary material for: High-efficacy and affordable hyperspectral pancreatic tissue image analysis using near-infrared spectroscopy
Source: J Pathol Inform. 2026 Mar 3;21:100651. doi: 10.1016/j.jpi.2026.100651 (PMC13054095; doi:10.1016/j.jpi.2026.100651)
Supplement: Supplementary material [file mmc1.pdf]

Supplementary Information

Towards High-efficacy and Affordable Hyperspectral Pancreatic Tissue Image Analysis using Near-Infrared Spectroscopy

Zheng Tang[a], Abhinav Mishra[e], Benjamin Mora[a], Bilal Al-Sarireh[b], Olivia Irvine[e], Brandon Mauri[a], Victoria Higginbotham[c], P M Anupama Bandaranayake[e], S. H. Chandrashekhara[d], Venkat Kanamarlapudi[c], and Debdulal Roy[e*]

[a]Department of Computer Science and Mathematics, Swansea University, SA2 8PP, UK
[b]Morriston Hospital, Heol Maes Eglwys, Morriston, SA6 6NL, UK
[c]Swansea Medical School, Swansea University Swansea SA2 8PP, UK
[d]All India Institute of Medical Sciences, Ansari Nagar, New Delhi- 110029, India
[e]Department of Chemistry, Swansea University, Swansea SA2 8PP, UK
[*]Deb.Roy@swansea.ac.uk

1 Data acquisition process in detail

Hyperspectral images were captured using the Hamamatsu InGaAs Camera with 640 (H) x 512 (V) effective number of pixels and 20 µm x 20 µm pixel size coupled to an Olympus IXplore IX73 standard inverted microscope. The NIREOS GEMINI interferometer was placed between the objective lens and the camera. A stabilized Globar IR Light Source from Thorlabs (SLS303) was used to illuminate the tissue samples using the microscope condenser system to achieve uniform illumination.

The images were cropped to 600 (H) x 450(V) pixels to match the optimum FOV of the interferometer while using the 4x objective lens which was focussed manually on the tissue samples. A hardware trigger was setup to synchronize image capture between the camera and the interferometer to acquire 245 images per tissue. Data were acquired as TIFF images using Hamamatsu HCIImageLive software and NI LabVIEW after selecting optimum exposure time. The corresponding FT parameters and position text files were also saved for each tissue.

2 High-resolution images of pancreatic cells and corresponding spectra

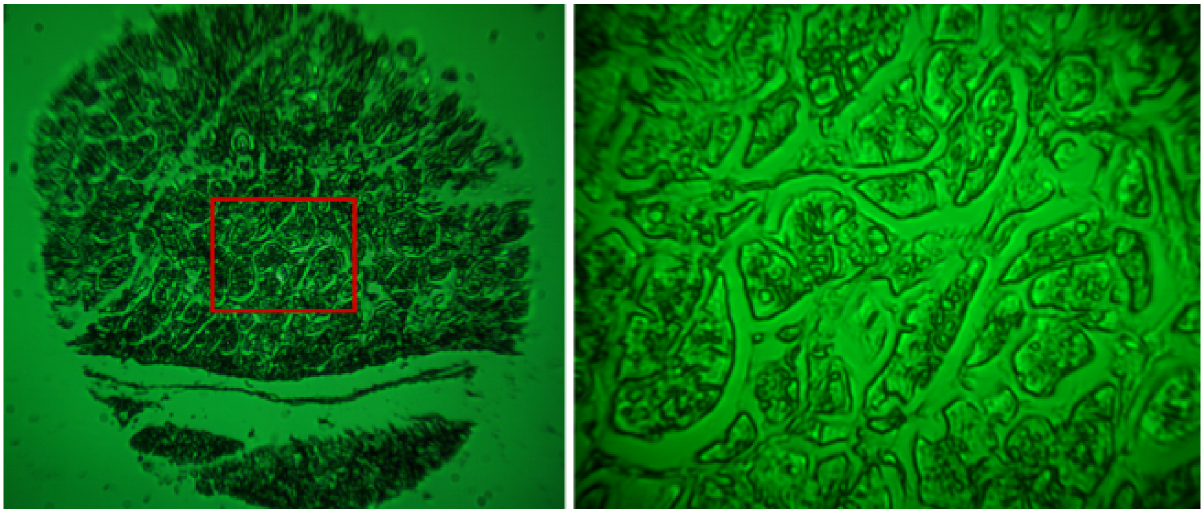

Fig. S 1. Left: Pancreatic tissue using 4x objective, Right: Zoomed-in image of the same tissue using 20x objective with FOV highlighted in red box on the left

Fig. S1 shows a high-resolution zoomed-in image of a particular area of pancreatic tissue with individual cells and its components visible.

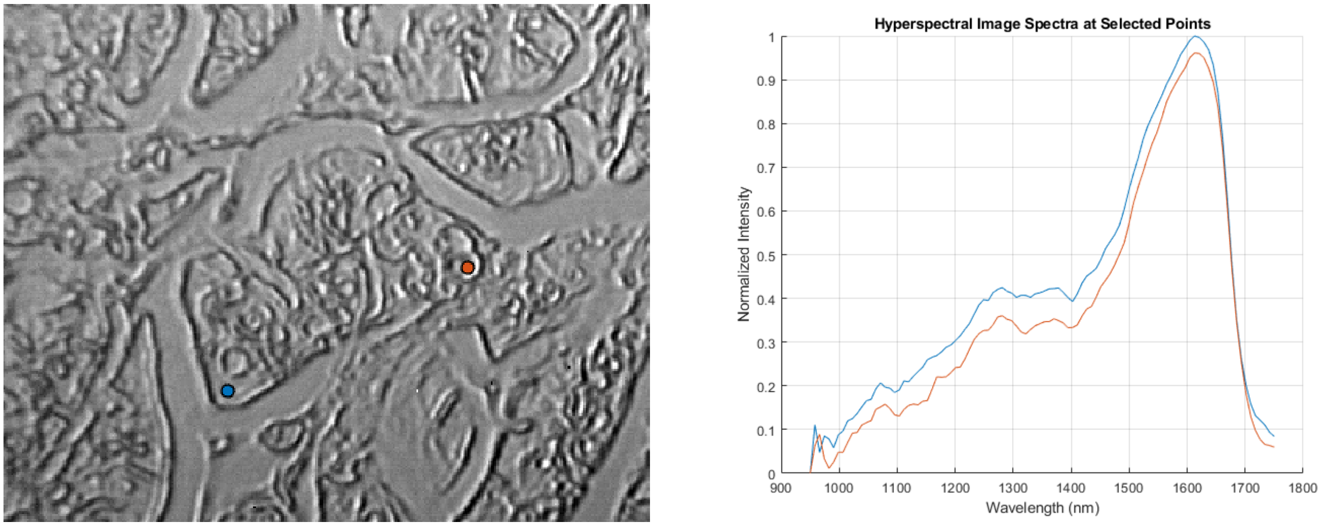

Fig. S 2. Left: Zoomed-in image of pancreatic cells. Right: Corresponding spectra of two points as shown in the image

Fig. S2 shows the spectra of two points in the image marked with blue and orange dots. The corresponding spectra from different regions of the cell show a common peak around 1620 nm with varying intensities, as shown on the y-axis.

3 Comparision with H&E images

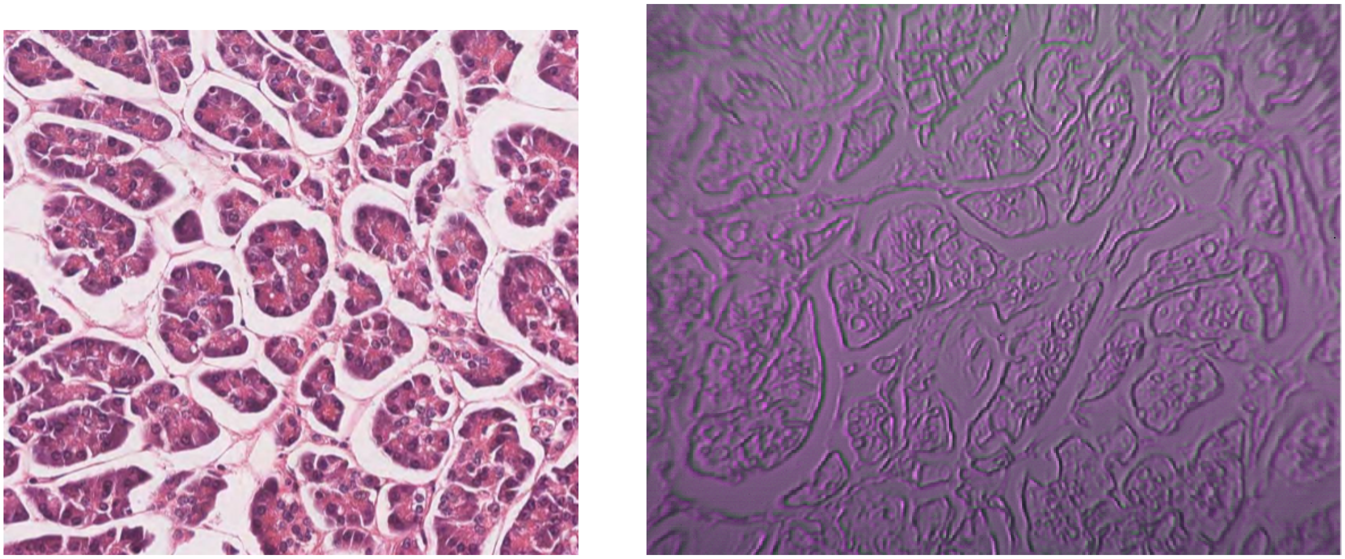

Fig. S 3. Left: H&E stained image of a similar pancreatic tissue from Tissuearray and Right: False-Colour stained image of pancreatic tissue using Hyperspectral imaging

4 Deep Learning Architecture and Implementation

4.1 Autoencoder Architecture for Hyperspectral Feature Learning

The autoencoder network was specifically designed to address the unique challenges of hyperspectral tissue analysis, where high-dimensional spectral information must be effectively compressed while preserving diagnostically relevant features. The encoder component utilizes a multi-scale convolutional approach that processes overlapping spatial patches extracted from

hyperspectral tissue images. Each patch undergoes successive convolutional layers with increasing filter complexity, where initial layers employ smaller kernels to capture fine-grained spectral features, while deeper layers use progressively larger kernels to integrate broader spectral patterns and spatial context information.

Batch normalization layers are strategically placed throughout the encoder to stabilize training dynamics, while dropout regularization prevents overfitting to the limited tissue dataset. The central bottleneck layer creates a compressed representation that distills complex spectral-spatial information into a lower-dimensional feature vector, balancing information preservation with computational efficiency. The decoder network mirrors the encoder architecture in reverse, reconstructing the original spectral patch through transposed convolutional layers. This reconstruction process serves as a self-supervised learning signal, forcing the encoder to learn meaningful spectral representations rather than arbitrary data transformations.

4.2 Patch-Based Processing and Feature Aggregation

The patch-based processing strategy addresses computational challenges while preserving local spatial relationships crucial for tissue characterization. The system extracts overlapping patches of fixed size using a sliding window approach, ensuring comprehensive coverage while maintaining spatial coherence. Each patch undergoes individual processing through the autoencoder, generating compressed feature representations that capture both spectral characteristics and local spatial organization.

The transformation from patch-level to sample-level representations employs multi-strategy aggregation combining several complementary statistical measures. Global mean aggregation captures average spectral-spatial characteristics, while maximum value aggregation identifies extreme spectral responses corresponding to specialized cellular structures or pathological regions. Standard deviation quantifies spectral-spatial heterogeneity within tissue samples, and interquartile range provides robust measures of spectral distribution spread less sensitive to outliers than standard deviation.

4.3 Classification Network Architecture and Optimization

The classification component employs a fully connected neural network optimized for aggregated hyperspectral tissue features. Single-layer architectures proved particularly effective, suggesting autoencoder-derived features require minimal additional transformation. The Exponential Linear Unit activation function emerged as optimal, providing improved gradient flow while maintaining computational efficiency and suitability for spectroscopic data where both positive and negative feature values carry discriminative information.

Dropout regularization is strategically applied to prevent overfitting while maintaining learning capacity, with rates tuned to balance adequate regularization against feature learning interference. Batch normalization layers stabilize training dynamics and improve convergence speed, maintaining consistent feature scales throughout the network and reducing sensitivity to initialization parameters.

4.4 Attention Mechanisms and Training Methodology

The attention mechanism provides interpretable insights by learning spatial importance weights indicating relative contributions of tissue regions to classification decisions. These attention maps highlight regions with high spectral variability or unusual tissue architecture, often corresponding to known pathological indicators and providing validation that the system focuses on clinically meaningful features.

The training process incorporates label smoothing for improved generalization, gradient clipping for stability, and cosine annealing learning rate schedules for adaptive parameter adjustment. Weight decay regularization prevents parameter overgrowth while early stopping based on validation performance prevents overfitting. This combination creates a robust optimization framework maximizing the network's ability to learn meaningful tissue characteristics from limited data while maintaining generalization capacity for clinical applications.
